# Configuration of Fibrous and Adipose Tissues in the Cavernous Sinus

**DOI:** 10.1371/journal.pone.0089182

**Published:** 2014-02-26

**Authors:** Liang Liang, Fei Gao, Qunyuan Xu, Ming Zhang

**Affiliations:** 1 Department of Anatomy, Capital Medical University, Beijing, China; 2 Department of Neurobiology, Capital Medical University, Beijing, China; 3 Department of Neurobiology, Capital Medical University, Beijing, China; 4 Department of Anatomy, University of Otago, Dunedin, New Zealand; Leiden University Medical Center, Netherlands

## Abstract

**Objective:**

Three-dimensional anatomical appreciation of the matrix of the cavernous sinus is one of the crucial necessities for a better understanding of tissue patterning and various disorders in the sinus. The purpose of this study was to reveal configuration of fibrous and adipose components in the cavernous sinus and their relationship with the cranial nerves and vessels in the sinus and meningeal sinus wall.

**Materials and Methods:**

Nineteen cadavers (8 females and 11 males; age range, 54–89 years; mean age, 75 years) were prepared as transverse (6 sets), coronal (3 sets) and sagittal (10 sets) plastinated sections that were examined at both macroscopic and microscopic levels.

**Results:**

Two types of the web-like fibrous networks were identified and localized in the cavernous sinus. A dural trabecular network constituted a skeleton-frame in the sinus and contributed to the sleeves of intracavernous cranial nerves III, IV, V_1_, V_2_ and VI. A fine trabecular network, or adipose tissue, was the matrix of the sinus and was mainly distributed along the medial side of the intracavernous cranial nerves, forming a dumbbell-shaped adipose zone in the sinus.

**Conclusions:**

This study revealed the nature, fine architecture and localization of the fine and dural trabecular networks in the cavernous sinus and their relationship with intracavernous cranial nerves and vessels. The results may be valuable for better understanding of tissue patterning in the cranial base and better evaluation of intracavernous disorders, e.g. the growth direction and extent of intracavernous tumors.

## Introduction

Various anatomical constraints, such as hard bones and cartilages, rigid ligaments and tendons, soft adipose and loose fibrous tissues, generate different pressures on the tumor surface and influence the direction, expansion, size, speed and morphology of tumor growth [1]. Such influence becomes particularly significant in the skull base where tumor growth is restricted in a very limited space by the cranial bones and dura.

The cavernous sinus or “lateral sellar compartment” [2] is one of the most complicated regions of the skull base and contains cranial nerves III, IV, V_1_, V_2_ and VI, the internal carotid artery, cavernous veins and adipose and fibrous tissues [3], [4], [5]. Tumors, such as meningiomas, schwannomas, chordomas and pituitary adenoma, may extend from or into the cavernous sinus [6], [7]. Three weak points of the sinus wall were identified for tumor invasion [5]. Although anatomy of the boundaries and neurovascular structures in the cavernous sinus has been extensively studied [8], [9], [10], [11], [12], [13], [14], [15], [16], [17], few studies have focused on the fibrous supporting matrix within the sinus [3], [4], [5], [10]. Understanding of configuration and localization of fibrous trabeculae and adipose tissue in the cavernous sinus may be valuable for better understanding of tissue patterning in the cranial base and better evaluation of intracavernous disorders, e.g. the growth direction and extent of intracavernous tumors [5], [18].

Technically, the study of adipose and loose fibrous tissues in the cadaver is complicated by the fact that great difficulties exist in dissecting out meticulous fibrous structures. Although histologic examination may be able to overcome the problem, the application of such method is greatly limited by the size of sample areas, alteration of tissue architecture during decalcification, and difficulty in tracing the origin of a fibrous structure. The newly developed anatomy technology, sheet plastination, not only preserves *in situ* positions of the cranial bones, meninges, nerves, vessels and loose fibrous tissue without decalcification but also allows these structures to be examined at both the macroscopic and microscopic levels [10], [19], [20]. The plastination process results in collagen fibers and neurofilaments being endogenously autofluorescent [20]. The purpose of this study was to combine the sheet plastination technique with confocal microscopy to reveal architecture and localization of intracavernous adipose and loose fibrous components and their relationship with intracavernous cranial nerves and vessels and cavernous meningeal wall.

## Materials and Methods

Nineteen cadavers (8 females and 11 males; age range, 54–89 years; mean age, 75 years) were used in this study.

### Ethics statement

The cadavers were bequeathed for medical education and research purposes and assigned to this project, and the written informed consent from the donor or the next of kin was obtained under the Human Tissues Act.

The cadavers were prepared as 19 sets of transverse (6 sets), coronal (3 sets) and sagittal (10 sets) plastinated sections. The thickness of the section was about 2.5–3.0 mm. Sheet plastination is a modern anatomical technique in which water and lipids of tissues and cells are replaced by curable and transparent resin. The plastination procedure was performed as previously described [19]. Four out of 19 cadaveric heads were pretreated with arachnoid staining and vascular and subarachnoid filling [19]. The prepared section was examined under a Leica MZ8 stereoscopic dissecting microscope (Leica, Heerbrugg, Switzerland). The high-resolution images of the selected areas were scanned and collected with an Epson Perfection V750 Pro Scanner (Epson, Jakarta, Indonesia) which scanning resolution was set up at 1200 dpi–6400 dpi.

The plastination process results in collagen fibers and neurofilaments being endogenously autofluorescent at the 488-nm excitation [20]. Differentiation among those autofluorescent fibers is based upon their morphology, fluorescent intensity and anatomical distribution. The plastinated section was observed under a Nikon AIR confocal laser scanning microscope (Nikon, Tokyo, Japan). The thickness of the optical section was set up at 16.7 µm under a 10× objective and the images were electronically recorded and montaged. A gender difference was not compared in this study.

## Results

**Figure 1:**
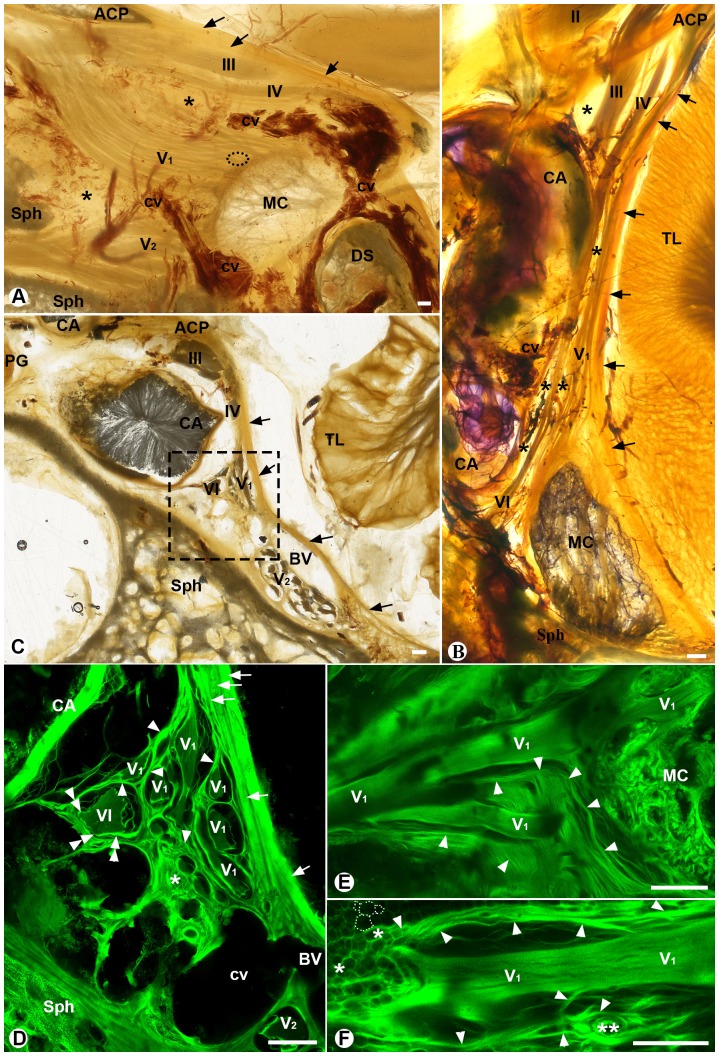
Sheet plastination sections of the cavernous sinus. **A, B** and **C** are the sagittal, transverse and coronal sections, respectively. **D, E** and **F** are the mirror confocal images of the selected areas (dashed-line boxes) of **A** and **C**. **A:** A sagittal section through the cavernous sinus at the level of the lateral edge of the dorsum sellae (DS). Arrows point to the dural roof of the cavernous sinus. Asterisks indicate the areas that are mainly occupied by adipose tissue and small cavernous veins (cv). **B:** A transverse section at the level of nerves V_1_ and VI. Arrows point the lateral meningeal wall of the sinus. Asterisks indicate the dumbbell-shaped adipose zone medial to intracavernous cranial nerves III, IV, V_1_ and VI. **C:** A coronal section at the middle level of the cavernous sinus. A cerebral bridging vein (BV) (see Figure 2D for its anterior segment) entered the sinus between the lateral meningeal wall (arrows) and nerve V_2_. **D:** The mirror confocal image of the dashed-line box in C, showing that the dural trabeculae (single arrowheads) originate from the medial laminae of the lateral dural wall (arrows) and encircle the branches of nerves V_1_ and VI, forming a dural trabecular network. Some dural trabeculae (double arrowheads) spirally encircle a nerve and contribute to the sleeve of the nerve. Asterisk indicates a fine trabecular network. **E:** The mirror confocal image of an area in the dashed-line circle in A, showing that some dural trabeculae (single arrowheads) from Meckel's cave (MC) longitudinally and loosely accompany a branch of nerve V_1_. **F:** The mirror confocal image of another area in the dashed-line circle in A, showing that the longitudinal dural fibers (single arrowheads) scatter and merge with the fine trabeculae in adipose tissue (asterisks). The dashed-line circles outline the basal membrane of some adipocytes. Double asterisks indicate a small vessel. ACP: anterior clinoid process; BV: cerebral bridging vein; CA: internal carotid artery; cv: cavernous veins; DS: dorsum sellae; MC: Meckel's cave; PG: pituitary gland; Sph: sphenoid bone; TL: temporal lobe; Cranial nerves II, III, IV, V_1_, V_2_ and VI; bars = 1 mm.

Cranial nerves III, IV, V_1_, V_2_ and VI passed through the cavernous sinus lateral to the internal carotid artery (Figure 1A). Meckel's cave occupied the posteroinferior part of the sinus and was posterior to nerves V_1_ and V_2_ (Figures 1A and 1B). Nerve VI traversed the sinus between the internal carotid artery and Meckel's cave posteriorly or nerve V_1_ anteriorly (Figures 1B and 1C). Among the nerves were loose connective tissue and small venous plexuses (Figures 1A and 1B). All the specimens observed in this study shown a similar anatomical pattern.

### Nature, architecture and localization of fibrous trabeculae in the cavernous sinus

Intracavernous nerves, arteries and veins were embedded in a web-like fibrous network. The network was composed of two groups of fibers that were termed as dural trabeculae and fine trabeculae in this study (Figure 1D). The majority of the dural trabeculae originated from the lateral dural wall and spirally encircled individual branches of the nerves (Figure 1D). Medially, they formed a continuous fibrous network around the vascular walls and connected with the endosteal dura of the sphenoid bone (Figure 1D). Other dural trabeculae originated from the dural penetration site of branches of a cranial nerve (Figure 1E). They longitudinally accompanied a nerve branch for a short distance (Figure 1E) and then scattered and disappeared in the fine trabecular network (Figure 1F). The fine trabeculae were those fibers in adipose tissue, e.g. basal membranes of adipocytes (Figure 1F). Thus, the dural trabeculae constituted a tough and strong fibrous skeleton-frame in the cavernous sinus and the fine trabeculae filled up spaces in the skeleton-frame, forming a base or matrix of the sinus.

**Figure 2:**
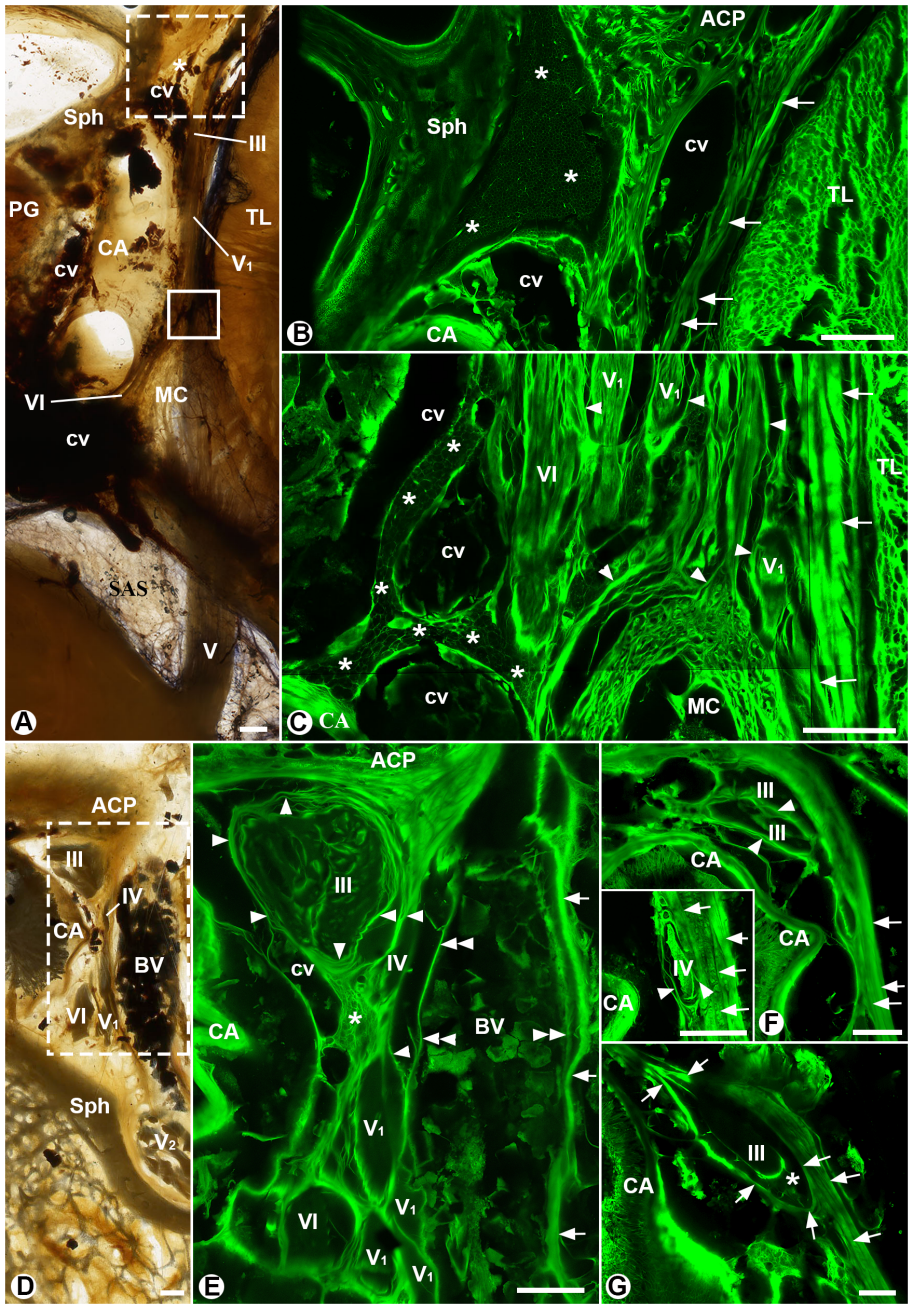
Localization of the adipose zone (A–C) and fibrous configuration of the sleeves of cranial nerves III and IV (D–G). **A:** A transverse section through the cavernous segment of the internal carotid artery (CA). **B:** The mirror confocal image of the dashed-line box in A, showing a large anterior end of the dumbbell-shaped adipose zone (asterisks) on the surface of the sphenoid bone (Sph), anterior to the internal carotid artery (CA) and its surrounding cavernous veins (cv), and posteromedial to the anterior clinoid process (ACP). Arrows point to the lateral meningeal dural wall of the sinus. **C:** The mirror confocal image of the solid line box in A, showing a small and irregular posterior end of the dumbbell-shaped adipose zone (asterisks) medial to the sleeves of the intracavernous cranial nerves VI and V_1_ and Meckel's cave (MC). Single arrowheads point to the dural trabeculae and arrows indicate multiple laminae of the lateral meningeal wall of the sinus. **D:** A coronal section at the level of the anterior clinoid process (ACP), about 6 mm anterior to Figure 1C. A cerebral bridging vein (BV) is located lateral to intracavernous cranial nerves III, IV, V_1_, V_2_ and VI. **E:** The mirror confocal image of the dashed-line box in D, showing that the dural trabeculae (single arrowheads) originate from the meningeal dura of the anterior clinoid process (ACP), encircle nerves III and IV and connect with the fine trabecular network (asterisk) and sleeves of nerves V_1_ and VI. Double arrowheads indicate the wall of the bridging vein. Single arrows point to the lateral wall of the cavernous sinus. **F** and **G:** The confocal images of two adjacent coronal sections about 6 mm (F) and 12 mm (G) posterior to E, showing that nerve III and its associated arachnoid cuff (asterisk) invaginate in the later wall (G) and then pieces the wall (F). The insert of F is from a different coronal section, showing that nerve IV pieces the lateral wall. Arrows point to the multiple laminae of the lateral dural wall of the cavernous sinus. Arrowheads point to the dural trabeculae originating from the medial lamina of the wall. ACP: anterior clinoid process; BV: cerebral bridging vein; CA: internal carotid artery; cv: cavernous veins; DS: dorsum sellae; MC: Meckel's cave; PG: pituitary gland; SAS: subarachnoid space; Sph: sphenoid bone; TL: temporal lobe; Cranial nerves III, IV, V_1_, V_2_ and VI; bars = 1 mm.

Although both dural and fine trabecular networks were connected with each other in the cavernous sinus, the bulk of the fine trabecular network or adipose tissue was distributed along the medial side of the intracavernous cranial nerves and shaped like a dumbbell (Figures 1B, 1D, 2A, 2B and 2C). The anterior end of the dumbbell-shaped adipose zone was large and located posteromedial to the anterior clinoid process, anterolateral to the internal carotid artery and its surrounding cavernous veins, and between the endosteal dura of the sphenoid bone and the intracavernous cranial nerves (Figures 2A and 2B). The posterior end of the adipose zone was small and irregular and located anteromedial to Meckel's cave and between the sleeves of cranial nerves VI and V_1_ and the internal carotid artery (Figures 2A and 2C). The bar of the dumbbell-shaped adipose zone was narrowed between the lateral wall of the internal carotid artery and the sleeves of nerve V_1_ and the lateral dural wall of the sinus (Figures 1B and 2A).

### Dural trabeculae and intracavernous cranial nerves

The sleeve or sheath of a cranial nerve in the cavernous sinus was mainly formed by the circular dural trabeculae and sporadically strengthened by those longitudinal dural trabeculae, particularly near the dura penetration point of the cranial nerve (Figures 1D and 1E). Several sleeves were often clustered together. The nerve branches in a cluster of the sleeves were not necessary from the same cranial nerve. As shown in Figure 1D, sleeves of some branches of nerve V_1_ and nerve VI were clustered together and anchored at the same site of the lateral dural wall. The sleeves of other V_1_ branches formed another cluster and anchored at a different site of the lateral dural wall. Between the clusters were those loose dural trabuculae, which provided a potential corridor to the adipose zone (Figures 1D and 2C).

The dural sleeves of branches of cranial nerves III and IV were loosely clustered together in the anterior part of the cavernous sinus (Figures 2D, 2E and 2F). They had a similar fibrous configuration to that of nerves V_1_ and VI. The sleeves were mainly formed by the circular dural trabeculae that originated from the meningeal dura of the anterior clinoid process (Figure 2E) and the lateral dural wall of the sinus (the insert of Figure 2F).

### The lateral dural wall of the cavernus sinus

The dura had an endosteal layer and a meningeal layer. The endosteal dura tightly invested the surface of the sphenoid bone and the meningeal layer formed the entire lateral wall of the cavernous sinus (Figures 1B and 1C). The meningeal dura of the lateral wall consisted of multiple densely packed fibrous laminae that had different sizes and ran in different directions (Figures 1D, 2C and the insert of 2F). The laminae were intermingled with each other, thus a cleavage plane between two adjacent laminae did not extend throughout the lateral wall (Figures 2C and the insert of 2F). The fibers from the most medial lamina of the lateral wall loosened, scattered and became part of the dural trabecular network (Figure 1D).

The lateral dural wall of the sinus was penetrated by cranial nerves III and IV and the cerebral bridging vein if it existed. The nerves III and IV and the associated arachnoid cuff gradually invaginated into the cavernous sinus (Figures 2E, 2F and 2G). The nerves and their branches were slung over by dural fibers rather than sandwiched in between two laminae of the dural wall (Figure 2G). At the dural entrance of a bridging vein, the vein ran between the lateral wall of the sinus and the sleeves of the cranial nerves (Figure 2D and 2E). The venous wall was extreme thin and fused with surrounding dural trabeculae, thus it is impossible to separate the lateral dural wall from the bridging vein at its dural entrance.

## Discussion

This study identifies the nature, fine architecture and localization of two types of the web-like fibrous networks in the cavernous sinus. A dural trabecular network forms a skeleton-frame in the sinus and contributes to the sleeves of intracavernous cranial nerves. A fine trabecular network, or adipose tissue, forms the matrix of the sinus and is mainly distributed along the medial side of the intracavernous cranial nerves, forming a dumbbell-shaped adipose zone.

### The adipose zone in the cavernous sinus

Since the pioneering work of Parkinson [2], it has been generally accepted that the cavernous sinus is an extradural compartment and contains not only arteries, nerves and venous plexus but also adipose tissue [3], [4]. Although the intracavernous neurovascular structures have been extensively studied [11], [13], [14], [21], [22], [23], [24], few studies have focused on the adipose and loose fibrous components within the sinus and their relationship with endosteal and meningeal dura. The present study found that connective tissue fibers of adipose tissue in the sinus formed a fine trabecular network which was mainly distributed in a 3-dimensional zone between the main vascular structures (e.g. the internal carotid artery and its surrounding cavernous venous plexus) and the intracavernous cranial nerves and shaped like a dumbbell. The dumbbell-shaped adipose zone is likely communicated with a similar fine trabecular network at the posteroinferior aspect of the pituitary gland, medial to the internal carotid artery [10].

### The dural trabecular network, cranial nerve sleeves and the lateral wall of the cavernous sinus

The nature of the lateral wall of the cavernous sinus and its relationship to the intracavernous cranial nerves have been a matter of debate for decades. Currently, there seems to be a consensus that the lateral wall of the cavernous sinus consists of an outer layer which is formed by the meningeal dura and an inner layer which is a thin semi-transparent membrane and contains the cranial nerves [4], [9], [16], [25], [26]. However, some controversies remain on the inner layer. Umansky and Nathan [27] described that the inner layer was formed by the sleeves of nerves III, IV, V_1_ and V_2_ with a reticular membrane extending between them. During dissection, they found that the inner layer was variable in its texture and morphological characteristics and in 28 out of 70 cadavers, the inner reticular membrane was incomplete. Dissections from Yasuda et al [16] and Campero et al [9] revealed that the inner layer was a continuation of the endosteal dura and invested the nerves running within the lateral wall. Some authors believe that the lateral wall of the cavernous sinus consists of more than two layers and cranial nerves run between the layers [25], [28].

The evidence from this study demonstrates that the lateral wall of the cavernous sinus is formed by the meningeal dura that consists of multiple densely packed fibrous laminae. The laminae of the lateral wall run in different directions and are intermingled with each other. There are some cleavages between the laminae but none of them extends throughout the whole wall. Dural fibers from the medial part of the lateral wall contribute to the web-like dural trabecular network in the cavernous sinus, but those dural trabeculae do not form an identifiable membrane-like structure lining on the medial surface of the lateral wall. The dural trabeculae encircle not only nerves III, IV and V but also nerve VI which is commonly believed to run within the sinus and does not contribute to the lateral wall. Thus, during dissection, the dural trabecular network together with parts of the medial laminae of the lateral wall may be misidentified as a complete or incomplete reticular inner layer of the lateral wall.

### The fine and dural trabecular networks and clinical implications

The complex configuration of the dural trabecular network and its relationship with the lateral wall and the fine trabecular network of the cavernous sinus may provide a better understanding of intracavernous disorders. For example, the pressure of various anatomical constraints affects strongly the direction, expansion, size, speed and morphology of tumor growth [1]. The characteristics of tumor growth in the cavernous sinus may be different between the fine and dural trabecular networks. Tumor growth in the dural trabecular network may theoretically encounter more mechanical resistance than that in the adipose zone [1] and more likely encase cranial nerves and dural trabeculae. As reported by Kawase et al [5], without sacrifice of the cranial nerves, full surgical removal of the tumor in this area was difficult and some microscopic tumor deposits often remained along the cranial nerve sleeves and dural trabeculae. In contrast, tumor growth in the adipose zone may have less mechanical resistance. Thus, tumors that extend from or into the cavernous sinus are more likely localized in and expanded along the dumbbell-shaped adipose zone. This hypothesis may warrant further clinical studies.

## Conclusions

This study identifies the nature, fine architecture and localization of two types of the web-like fibrous networks in the cavernous sinus. The dural trabecular network forms a skeleton-frame in the sinus and contributes to the sleeves of intracavernous cranial nerves. The fine trabecular network forms the matrix of the sinus. Precise configuration of the intracavernous fibrous networks and their relationship with intracavernous cranial nerves and vessels and meningeal wall may be valuable for better understanding of tissue patterning in the cranial base and better evaluation of intracavernous disorders, e.g. the growth direction and extent of intracavernous tumors.
